# GenIce: Hydrogen‐Disordered Ice Generator

**DOI:** 10.1002/jcc.25077

**Published:** 2017-10-12

**Authors:** Masakazu Matsumoto, Takuma Yagasaki, Hideki Tanaka

**Affiliations:** ^1^ Division of Superconducting and Functional Materials Research Institute for Interdisciplinary Science, Okayama University Okayama 700‐8530 Japan

**Keywords:** hydrogen‐disordered ice, lattice generator, ice polymorphs, clathrate hydrates, zeolite

## Abstract

GenIce is an efficient and user‐friendly tool to generate hydrogen‐disordered ice structures. It makes ice and clathrate hydrate structures in various file formats. More than 100 kinds of structures are preset. Users can install their own crystal structures, guest molecules, and file formats as plugins. The algorithm certifies that the generated structures are completely randomized hydrogen‐disordered networks obeying the ice rule with zero net polarization. © 2017 The Authors. Journal of Computational Chemistry Published by Wiley Periodicals, Inc.

## Introduction

Water has a complex phase diagram as a single component small molecule. At least 17 ice polymorphs have been discovered so far.^1^, ^2^, ^3^, ^4^, ^5^, ^6^ There are two reasons for this diversity of ice phases. One is that water is a network‐forming substance linked by hydrogen bonds, and the other is hydrogen‐disorder.

All the ice phases obey the ice rule of Bernal–Fowler^7^ (except for amorphous ices and ultra‐high pressure ice X). This rule requires that each water molecule accepts and donates two hydrogen bonds. Under this condition, a water molecule takes one of six different orientations. In many ice structures, the energies of the six molecular orientations are considerably close to each other and can be regarded as degenerated. This results in the residual entropy (Pauling's entropy) at low temperature.^8^ We refer to such ice as hydrogen‐disordered ice. Occurrence of residual entropy is one of distinguishing characteristics of ice.

A hydrogen bond network can be regarded as a directed graph. In graph theory terminology, an ice hydrogen bond network is a 2‐regular directed graph (each vertex has two incoming edges and two outgoing edges) if it obeys the ice rule.

Water molecules also form clathrate hydrates and filled ices. They are solid solutions of water and hydrophobic small molecules.^9^ If guest molecules can be removed from clathrate hydrate or filled ice by some means, a new ice phase may be obtained. For example, ice XVI is obtained from CS‐II neon hydrate by vacuum pumping.^5^ In this case, the ice phase and the clathrate hydrate phase share the same network skeleton structure. A similar relationship is seen between cubic ice I and filled ice C_2_,^10^ ice II and filled ice C_1_,^11^ and ice XVII and filled ice C_0_.^6^ Thus, the diversity of ice crystals and the diversity of clathrate hydrate structures are closely related.

It is necessary to prepare a large number of properly hydrogen‐disordered arrangements of ice to calculate its properties in computer simulations. In this article, we introduce an open source software, GenIce, which enables one to generate homogeneous and sufficiently randomized hydrogen‐disordered ice structures at high speed. GenIce is designed to be extended easily by installing plugins for lattices, molecules, and output formats. See Appendix how to obtain the software.

## Methods

Stillinger first introduced an algorithm for making hydrogen‐disordered ice structures.^12^ The initial structure is a hydrogen‐ordered ice with no net polarization. The algorithm searches for unidirectional cyclic paths (homodromic cycles) of hydrogen bonds by random walk on the network and introduces randomness to the network by reversing the cyclic paths one after another. In this algorithm, the search stops when the random walk intersects its own trajectory. This makes a small cyclic path and, therefore, the algorithm is called the short‐loop algorithm.^13^

Barkema proposed a different method.^13^ In this algorithm, first, a structure that completely satisfies the ice rule is prepared, and a H_3_O^+^ ion and an OH^–^ ion are generated by moving a hydrogen atom along the hydrogen bond. Next, a hydrogen atom of the H_3_O^+^ ion is moved along the hydrogen bond to transfer the protonic defect by the Grotthuss mechanism. The random proton transfer is continued until the protonic defect reaches the OH^‐^ ion generated initially. Since a long distance transfer of H_3_O^+^ is required to annihilate the defect pair, this algorithm is called the long‐loop algorithm.

Both of these algorithms start from a hydrogen‐ordered structure and invert the orientation of the hydrogen bonds randomly to obtain a hydrogen‐disordered structure. These methods may yield a nonisotropic and nonuniform structure due to improper selections of the initial configuration and insufficient randomization. Therefore, separate tests are necessary to certify that the generated structure is indeed sufficiently disordered.

Unlike the conventional methods, GenIce uses totally randomized structures as initial states.^14^ An initial random structure is converted so that it obeys the ice rule (step 1). Then, the net polarization is removed by an efficient algorithm which can be used for large systems (step 2).

### Step 1: Ice rule

We first generate a four‐regular undirected graph based on the hydrogen bond network topology of the ice phase, and make a directed graph from the undirected graph by replacing undirected edges with randomly oriented directed edges. Since the obtained randomly directed graph does not satisfy the ice rule, defects are purged by the following algorithm.

1. Push all vertices that do not have two incoming bonds, which correspond to defects in ice, into a FIFO (first‐in, first‐out) queue.
2. Pop a vertex from the queue and count the number of incoming bonds for the vertex, *N_i_*.
3. If *N_i_* is less than 2, choose an outgoing edge of the vertex randomly. If *N_i_* is greater than 2, choose an incoming edge of the vertex randomly. Otherwise, go to 7.
4. Invert the chosen edge.
5. If the original vertex is still a defect, push it back to the queue.
6. If the neighbor vertex becomes a defect by the inversion, push it to the queue.
7. Go to 2 unless the queue is empty.

Essentially, this algorithm is similar to the procedure proposed by Buch.^14^

### Step 2: Removal of the net polarization

The procedure in step 1 certifies the uniformity of the obtained hydrogen bond network. However, usually, there remains a nonzero net polarization in the resultant hydrogen bond network. A component of the net dipole vector parallel to the direction of one of the primitive translational vectors, *a*, *b*, or *c*, is always an integral multiple of a certain constant value for the hydrogen bond network that obeys the ice rule under periodic boundary conditions, and the polarization can be removed only by inverting homodromic cycles of hydrogen bonds spanning the cell. Random inversion of homodromic cycles is a common method to introduce randomness to hydrogen bond networks so as not to violate the ice rule. However, a randomly generated homodromic cycle hardly spans the cell neither by the short‐ nor by the long‐loop algorithm when the system is large. Therefore, more efficient algorithm is necessary to find cell‐spanning homodromic cycles.

In GenIce, the following algorithm is employed to remove the net polarization efficiently. Here, we explain the way to reduce the dipole moment along the *c* axis as an example.

1. Choose a vertex of the network, say *i*, randomly.
2. When the fractional coordinate of a given point in the cell is written as (*a*,*b*,*c*), the *c*‐antipode of the given point is (*a*,*b*,*c*+0.5). Find the vertex, say *j*, that is closest to the *c*‐antipode of *i*.
3. The shortest path from *i* to *j* in the directed graph is obtained by Dijkstra's algorithm.^15^
4. The shortest path from *j* to *i* is obtained in the same manner.
5. Merge the two paths to make a cycle. It can be a homodromic cycle spanning the cell along the c‐axis.
6. If the polarization along the c axis decreases by inverting the homodromic cycle, invert it. Otherwise, go back to 1.
7. Repeat inversion of homodromic cycles until the net polarization vanishes.

The procedure is applied to the *a*, *b*, and *c* axes of the cell independently. The processing time depends on the initial configuration, but it is almost linear against the number of molecules in the simulation cell for practical system sizes (up to the tens of thousands of molecules).

### Typical usage of GenIce

To create a 3 × 3 × 3 unit cell replica of hydrogen‐disordered ice IV (4) in the Gromacs format with the TIP4P water model, enter the following command:

genice 4 ‐‐water tip4p ‐‐rep 3 3 3 > ice4.gro

See Table 1 for the preset ice structures. Note that the same structure may have different names in different structure frameworks. They are listed in Table 2. One can make a new ice structure by preparing a lattice plugin.

**Table 1:** The list of available ice types.

Symbol	Description
1h, 1c	Most popular Ice I (hexagonal or cubic).
2	Hydrogen‐ordered ice II.
2d	Hypothetical hydrogen‐disordered counterpart of ice II.^16^
3, 4, 5, 6, 7, 12	Conventional high‐pressure ices III, IV, V, VI, VII, and XII.^17^
16	Negative‐pressure ice XVI.^5^
17	Negative‐pressure ice XVII.^6^
0	Hypothetical ice “0”.^18^
I	Hypothetical ice “*i*” = Zeolite BCT.^19^
C0‐II	Filled ice C_0_ (Alias of 17).^20^
C1	Filled ice C_1_ (Alias of 2).^11^
C2	Filled ice C_2_ (Alias of 1c).^10^
sTprime	Filled ice “sT'”.^20^
CS1, CS2, CS4, TS1, HS1, HS2, HS3	Clathrate hydrates in the Kosyakov's nomenclature.^21^
sI, sII, sIII, sIV, sV, sVII, sH	Clathrate hydrates in the Jeffrey's nomenclature.^22^
MTN	Alias of ice XVI.
RHO	Hypothetical ice at negative pressure, ice “sIII”.^23^
FAU	Hypothetical ice at negative pressure, ice “sIV”.^24^
CRN1, CRN2, CRN3	4‐coordinated continuous random networks, models for low density amorphous ice.^25^
Struct01 – Struct84	Space Fullerenes.^26^
A15, sigma, Z, mu, zra‐d, C36, C15, C14, delta, psigma FK9layers, Hcomp, FK6layers	Space Fullerenes (Aliases). See the data source for their names.^26^
T	Dual of the Frank–Kasper structure T.^26^ “II+IV a” structure.^27^

It will be updated over time (see the latest online manual). Ice names with double quotations have not been verified experimentally.

**Table 2:** Correspondence between structure frameworks.

Frank–Kasper dual^26^, ^28^	Ice	Clathrate hydrates		Zeolites^29^	Filled ices
		Jeffrey^22^	Kosyakov^21^		
A15	–	sI	CS1	MEP	–
C15	16	sII	CS2	MTN	sII
sigma	–	sIII	TS1	–	–
Z	–	sIV	HS1	–	–
C14	–	sV	HS3	–	–
–	–	sVII	CS4	SOD	–
–	–	sH	HS3	DOH	–
–	17	–	–	–	C0
–	2	–	–	–	C1
–	1c	–	–	–	C2

Different hydrogen order can be obtained by specifying the random seed with the ‐s option.

genice 4 ‐s 1234 ‐‐water tip4p ‐‐rep 3 3 3 > ice4.gro

By default, the hydrogen bonds are appropriately arranged to make the polarization be zero. The option ‐‐nodep avoids the depolarization process.

The density of ice is set automatically according to the shortest distance between water molecules. It can be specified by the ‐‐dens x option where x is the density of water in ice given in g cm^−3^ (the mass of guest molecules is not included for clathrate hydrates and filled ices).

**Figure 1:**
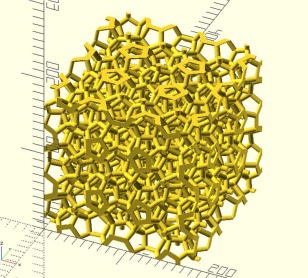
Unit cell of space fullerene type T rendered in the OpenSCAD software. OpenSCAD produces files in the STL format which is a standard format for typical 3D printers. Space fullerene type T is one of the most complex ice‐like structures ever predicted; its unit cell consists of 920 water molecules. [Color figure can be viewed at wileyonlinelibrary.com]

The output style is specified with the ‐‐format option. Several output formats are available and they are listed in Table 3. The default output format is the Gromacs .gro format. GenIce can output .scad files for the OpenSCAD software which can generate input files for 3D printers. Figure 1 shows the unit cell of space fullerene type T rendered by the OpenSCAD software.

**Table 3:** List of output types.

Symbol	Application	File suffix	Water^a^	Guest^a^	HB
g, gromacs	Gromacs^b^	.gro	A	A	None
d, digraph	Topology of the HB network.	.ngph	None	None	O
y, yaplot	YaPlot^c^	.yap	A	A	O
o, openscad	OpenSCAD^d^	.scad	C	None	O
p, python	Python	.py	C	None	None
cif, cif2	CIF file	.cif	A	A	None
xyz	XYZ file	.xyz	A	A	None

a A: Atomic positions, C: Center of mass (Oxygen position).

b http://www.gromacs.org

c https://github.com/vitroid/Yaplot.

d http://www.openscad.org.

genice T ‐‐rep 1 1 1 ‐‐format o > T.scad

One can also prepare clathrate hydrates filled with guest molecules. For example, to stuff CO_2_ molecules in all “12” cages (5^12^) and 50% of “14” cages (5^12^6^2^) of CS1 hydrate, add the “‐g” options as follows:

genice CS1 ‐g 12=co2 ‐g 14=co2*0.5 > cs1.gro

The option also accepts compositions for mixed hydrates. The syntax is (cage type)=(guest name)*(fill ratio)+(guest name)*(fill ratio)+…, for the ‐g option.

genice ‐g 16=me*0.5+co2*0.3 ‐g 12=me* 0.9 CS2 > cs2.gro

There are only a few types of guest molecules prepared by default. They are listed in Table 4. One can define new molecular types by writing molecule plugins.

**Table 4:** Types of preset guest molecules.

Symbol	Type
co2	CO_2_
uathf	United atom 5‐site tetrahydrofuran.
me	United atom monatomic methane.
g12, g14, g15, g16	A monatomic dummy site.
empty	Leave cages empty.

Designing semiclathrate hydrates is complicated. Refer to the manual.

## Plugins

GenIce offers extensions by plugins. There are three types of plugins: molecular plugin, lattice plugin, and format plugin. Users can install their own plugins to serve new kind of guest and water molecules, crystal lattices, and output formats for specific applications.

## Incorporating Zeolite Structures

It is possible to make ice structures from porous SiO_2_ zeolite frameworks. Such structures can be candidates for stable ice phases under negative pressure because of the low density.^5^, ^23^, ^24^ We provide the cif2ice tool (https://github.com/vitroid/cif2ice). This tool obtains CIF files of zeolites from the zeolite database web site^29^ and makes lattice modules for GenIce.

## Summary

We introduce a tool for generating hydrogen‐disordered ice structures, GenIce. The algorithm employed in GenIce certifies the uniformity of hydrogen‐disorder and the zero net polarization. Various ice and clathrate hydrate structures are preset. Users can install new host (water) molecules, guest molecules, lattice structures, and output formats as plugins.

The uniformity of hydrogen‐disorder may affect the quality and reliability of computer simulations of ices and clathrate hydrates. Nowadays, the system size of computer simulations is getting larger and larger, and there is a great need for preparing many hydrogen bond networks of different orderliness efficiently. GenIce has already been used in our researches,^16^, ^30^, ^31^, ^32^, ^33^, ^34^ and we will continue to improve the tool to generate more complex structures such as ice including various types of defects which play important roles in dynamic properties.^35^

## Appendix

GenIce is served at the GitHub (https://github.com/vitroid/GenIce) as an open source software since 2015. Feedbacks, proposals for improvements and extensions, and bug fixes are sincerely appreciated. Developers and test users are also welcome. The latest users' manual is available at the website. GenIce is written in Python 3, and can be obtained as the “genice” package from the PyPI (Python Package Index) repository using the easy_install or pip commands. Extension plugins should also be written in Python 3.
